# The project to understand and research preterm pregnancy outcomes and stillbirths in South Asia (PURPOSe): a protocol of a prospective, cohort study of causes of mortality among preterm births and stillbirths

**DOI:** 10.1186/s12978-018-0528-1

**Published:** 2018-06-22

**Authors:** Elizabeth M. McClure, Sarah Saleem, Shivaprasad S. Goudar, Sangappa Dhaded, G. Guruprasad, Yogesh Kumar, Shiyam Sunder Tikmani, Masood Kadir, Jamal Raza, Haleema Yasmin, Janet L. Moore, Jean Kim, Carla Bann, Lindsay Parlberg, Anna Aceituno, Waldemar A. Carlo, Robert M. Silver, Laura Lamberti, Janna Patterson, Robert L. Goldenberg

**Affiliations:** 1 RTI International, 3040 Cornwallis Road, Durham, NC 27709 USA; 2 Aga Khan University, Karachi, Pakistan; 3 KLE Academy of Higher Education and Research’s, J N Medical College, Belagavi, Karnataka India; 4 Bapuji Educational Association’s J.J.M. Medical College, Davangere, Karnataka India; 5 National Institute of Child Health, Karachi, Pakistan; 6 Jinnah Postgraduate Medical Centre, Karachi, Pakistan; 7 University of Alabama at Birmingham, Birmingham, AL USA; 8 University of Utah School of Medicine, Salt Lake City, UT USA; 9 Bill & Melinda Gates Foundation, Seattle, WA USA; 10 Columbia University, New York, NY USA

**Keywords:** Preterm birth, Stillbirth, Mortality, South Asia

## Abstract

**Background:**

In South Asia, where most stillbirths and neonatal deaths occur, much remains unknown about the causes of these deaths. About one-third of neonatal deaths are attributed to prematurity, yet the specific conditions which cause these deaths are often unclear as is the etiology of stillbirths. In low-resource settings, most women are not routinely tested for infections and autopsy is rare.

**Methods:**

This prospective, cohort study will be conducted in hospitals in Davengere, India and Karachi, Pakistan. All women who deliver either a stillbirth or a preterm birth at one of the hospitals will be eligible for enrollment. With consent, the participant and, when applicable, her offspring, will be followed to 28-days post-delivery. A series of research tests will be conducted to determine infection and presence of other conditions which may contribute to the death. In addition, all routine clinical investigations will be documented. For both stillbirths and preterm neonates who die ≤ 28 days, with consent, a standard autopsy as well as minimally invasive tissue sampling will be conducted. Finally, an expert panel will review all available data for stillbirths and neonatal deaths to determine the primary and contributing causes of death using pre-specified guidance.

**Conclusion:**

This will be among the first studies to prospectively obtain detailed information on causes of stillbirth and preterm neonatal death in low-resource settings in Asia. Determining the primary causes of death will be important to inform strategies most likely to reduce the high mortality rates in South Asia.

**Trial registration:**

Clinicaltrials.gov (NCT03438110) Clinical Trial Registry of India (CTRI/2018/03/012281).

## Background

Neonatal mortality is common in South Asia and sub-Saharan Africa with rates as high as 40 to 50 per 1000 live births in some countries compared to rates as low as 2 per 1000 live births in Scandinavia [1, 2]. Worldwide, at least 2.6 million neonatal deaths occur annually, with more than one-third attributed directly to preterm birth [3]. Globally, the risk of death from preterm birth is highest in South Asia and sub-Saharan Africa [4]. Although mortality rates are often higher in Africa, numerically, more infants die in South Asia. Preterm neonates die from prematurity-related complications including respiratory distress syndrome (RDS), necrotizing enterocolitis (NEC), and intraventricular hemorrhage (IVH), as well as asphyxia, infection, and congenital anomalies, conditions not always attributed to preterm birth [4]. However, few cause of death studies—especially in low-resource settings in low and middle-income countries (LMIC)—have determined specific causes of preterm death, instead attributing all neonatal deaths of infants < 37 weeks to prematurity. Furthermore, little is known about causes of death among stillbirths in LMIC and especially specific types of infections associated with stillbirth.

One of the important goals of international organizations is to reduce neonatal mortality in LMIC, with recent efforts highlighting the importance of improving survival of preterm infants [5, 6]. Gaps in our knowledge of the medical conditions that increase risk of death in preterm infants and the circumstances of these deaths have impeded progress to improve survival. Determining major infectious and pathological causes, as well as clinical events that contributed to the death is necessary not only to understand the cause of death in preterm infants, but also to identify effective treatments to improve survival.

Less is known about causes of stillbirth than neonatal mortality. Globally, the highest reported rates of stillbirth occur in Pakistan. In most countries, stillbirth rates are equivalent to or greater than neonatal mortality rates with about 3 million third trimester stillbirths occurring annually. In high-income countries, 50% of stillbirths occur prior to 28 weeks and fully 80% occur prior to term [7]. The percent of stillbirths occurring in the preterm period in LMIC is unknown. Thus, we estimate that most perinatal mortality in LMIC occurs in infants born preterm.

Stillbirths are caused by a variety of maternal and fetal conditions, including placental abruption, obstructed labor, preeclampsia, placental insufficiency, infection, congenital anomalies and cord complications, conditions that also contribute to neonatal mortality. The distribution of these causes and the sequence of events leading to stillbirth in LMIC are generally unknown. One study suggests that when assessing preterm birth, the true picture of preterm birth may be obscured if stillbirth is excluded [8]. Thus, given the substantial overlap in etiologies between preterm neonatal deaths and preterm stillbirths, and the large contribution of stillbirths to the preterm birth rate, evaluating causes of death among stillbirths will substantially increase our understanding of all preterm deaths.

For both neonatal deaths and stillbirths, infections are often not identified and have largely been under-reported in low-resource settings where both logistics and lack of appropriate technology may limit investigations into infections. Over 100 organisms have been reported to potentially cause stillbirth or neonatal death [9]. Since the identification of pathogens responsible for fetal or neonatal death may not be obtained from blood cultures alone, the identification process becomes more complicated with testing required of specific tissues such as the placenta, and fetal or neonatal organs, often with molecular assays.

In many regions in South Asia, most deliveries now occur in health facilities [10]. Despite the dramatic increase in hospital deliveries in the last decade in this region, little reduction in neonatal mortality or stillbirth has been realized. Determining the main causes and risk factors for perinatal mortality will ultimately inform potential strategies to reduce the high neonatal mortality and stillbirth rates currently seen in South Asia.

## Methods and design

Our primary objectives are to determine the primary causes of death among preterm live-born infants and stillbirths in South Asia (Pakistan and India) including determining the main causes of death for preterm infants and stillbirths who are delivered in selected hospitals in India and Pakistan. Secondary objectives include determining the proportion of stillbirths and neonatal deaths caused by infection. For stillbirth and preterm deaths caused by infections, we will also determine the proportion of deaths caused by specific pathogens of interest. We also aim to identify maternal and obstetric risk factors which contribute to preterm deliveries that result in stillbirth and neonatal death. Finally, we will determine practices in the obstetric unit and neonatal intensive care unit (NICU) associated with risk of stillbirth or neonatal death and evaluate potential preventability of these preterm infant deaths and stillbirths.

### Design

This prospective, observational cohort study of pregnant women will evaluate causes of stillbirth and neonatal death (≤ 28 days) among preterm births. Women who deliver either a preterm live birth, 20–37 weeks calculated based on a predefined algorithm, [11, 12] or stillbirth ≥ 20 weeks at any gestational age in a study hospital will be enrolled. Among infants admitted to the hospital, the clinical care, evaluation and results of any investigations will be documented with additional, prospectively defined research investigations. Specifically, the placenta for all deliveries will be collected for evaluation and minimally invasive tissue sampling (MITS) as well as standard autopsy will be conducted on both stillbirths and neonatal deaths with additional consent. Tissues obtained will be evaluated histologically and tested by PCR for organisms of interest. Among infants not admitted who ultimately die at home, verbal autopsy performed by a trained health worker will be collected. All information will be collated to help inform the determination of cause of death. The final primary cause of death and contributing fetal/neonatal and maternal factors will be determined by a panel of experts using all available information and pre-defined guidance.

### Study population

The study will be conducted at 5 to 6 hospitals in Karachi, Pakistan and in Davangere, India. All women who deliver at a study hospital in one of these sites will be screened for study eligibility.

#### India

Study recruitment will be conducted in Davangere, India, located in central Karnataka in southern India. In Davangere, the 3 hospitals include both public and private services which together cover the catchment region surrounding Davangere and reflect the population of the Karnataka State of India. Altogether, these hospitals have approximately 16,000 annual deliveries, with an estimated preterm delivery rate of approximately 30%. Across the three hospitals, the stillbirth rate is about 25 per 1000 deliveries.

#### Pakistan

Study recruitment will be conducted in Karachi, Pakistan, located in southern Pakistan. Pregnant women will be recruited in the obstetrics department in the Jinnah Post-graduate Medical Centre (JPMC) and infants followed at the neonatal intensive care unit (NICU) at the adjacent National Institute of Child Health (NICH), which accepts referrals from JPMC. These hospitals serve a wide range of the population to provide advanced care of neonates in Karachi. JPMC has 15,000 annual deliveries with approximately 35% preterm births. In addition, the stillbirth rate is estimated at 35 per 1000 deliveries. Other hospitals may be included as necessary.

### Study procedures

#### Initial screening

Potential study participants will be recruited from study hospitals in labor and delivery. Upon identification, a brief assessment of the woman’s eligibility will be made, which will include her estimated gestational age and the status of the fetus.

### Inclusion and exclusion criteria

#### Inclusion criteria

- ■ Pregnant women ≥ 14 years of age.
- ■ Women who are admitted with an imminent preterm delivery based on clinical indications (20–37 weeks gestation) or women who have a known stillbirth (intrauterine fetal death ≥ 20 weeks).

#### Exclusion criteria

- ■ Induced (medical) abortion (< 20 weeks)
- ■ For live births, Unable to determine the gestational age at delivery

### Ethics and consent

Before a woman participates in any research activity, research staff will obtain her informed consent to voluntarily participate in the study. Research staff will give adequate opportunity to each potential participant and/or immediate family members to read the consent form and ask questions. Recognizing the range of literacy levels in study catchment regions, the consent process will include a verbal review and explanation of the study procedures and consent form. If a woman has inadequate time to review the study and consent prior to delivery, consent may be requested following her delivery. Families for which a stillbirth or neonatal death occurs will have additional consent requested for additional investigations; a team from the hospital will provide the family with information about the results of relevant tests and circumstances around their infant’s death or stillbirth.

The study will be overseen by ethics review committees at the Aga Khan University (Karachi, Pakistan), KLE Academy of Higher Education and Research (Belagavi, India), J.J.M. Medical College (Davangere, India), and RTI International. Only women with informed consent will be enrolled in the study.

#### Gestational age dating

To be included in the study, a woman either delivers a stillbirth (≥ 20 weeks gestation) or a preterm (< 37 weeks) live birth. Gestational dating will be done using the hierarchy of methods established by the American College of Obstetrics and Gynecology [11, 12], using report of reliable last menstrual period and ultrasound examinations. If neither is available, the Ballard examination (for preterm live births) will be used.

#### Minimally invasive tissue sampling (MITS)

MITS, a relatively new technique which uses needle biopsies of selected organs to obtain tissue samples for histology and PCR for organism identification has been proposed as an alternate method to full autopsy for providing information on cause of death [13]. In this study, consent for MITS will be obtained for both stillbirths and neonatal deaths, in addition to full autopsy, or in cases wherein the family declines autopsy.

### Enrollment procedures

#### Overview

Following confirmation of eligibility and attainment of signed informed consent, clinical and research investigations will be collected as summarized in Table 1. Maternal assessments a rectovaginal swab will be obtained from each pregnant woman for assessment of group B streptococcus (GBS). In addition, each participant’s clinical status will be assessed, including a physical examination, medical and obstetric history, and review of procedures for current delivery. Immediately following delivery, the placenta and cord blood will be collected.

**Table 1:** Types of information collected for preterm infants enrolled, neonatal deaths and stillbirths and for NICU admissions

	All Enrolled Infants	Infant Deaths ≤ 28 days and Stillbirths	Infants admitted to NICU
General	• GA dating by LMP and US or Ballard examination • Obstetric history • Delivery information	• Autopsy • MITS: brain, liver, blood and lung tissue samples • X-ray • Placental pathology • Cord blood • Storage of samples for quality control • External physical examination • Photograph	• Complete blood count • CRP • Random blood sugar • Temperature • Head ultrasound when clinically indicated • Chest and abdominal x-ray when clinically indicated • Culture of blood, CSF and others when clinically indicated
Infection study	• Maternal GBS swab on hospital admission • Maternal syphilis screening test • Maternal serum for antibody analysis	• Placental tissue (PCR) • Liver, lung and brain tissue (PCR) • Cord and/or heart blood (PCR) For all deaths, we will collect several fetal/infant dried blood spot cards (DBS) to be used for organism confirmation if necessary	
Hemoglobin, metabolic and genetic disorders		Testing for cause of death using DBS, blood and tissue DNA, if indicated based on screening criteria	

#### Stillbirth assessment

Stillbirths will have a complete assessment including a physical examination following delivery, photograph and x-rays (as appropriate). With consent, MITS as well as an autopsy will be performed. In addition, the placenta will be examimed grossly and histologically and placental specimens as well as cord blood samples collected.

#### Newborn assessment

Newborns will have a physical assessment completed as soon as possible following delivery, including confirmation of gestational age. If the clinical examination suggests that the prior estimation of gestational age is unreliable, the clinician may exclude term infants following delivery. All preterm infants enrolled in the study will be followed through 28 days of life to assess outcomes, regardless of their admission to the NICU. Among those infants admitted to participating study NICUs, their clinical care, clinical investigations, and any conditions diagnosed during the NICU admission will be documented. Among those infants discharged alive prior to or on day 28, a follow-up visit will be made to assess the infants’ status at 28 days. For all live-born preterm infants, the placenta will be evaluated grossly. A placental specimen will be collected for potential PCR analysis and the placenta placed in formalin for potential histological analysis in case of death. In addition, cord blood will be collected for all births for potential PCR and antibody analyses as well as other assays.

#### Neonatal death assessment

For those who die within 28 days, a complete assessment of circumstances surrounding their death including the clinical summary, an autopsy and/or MITS (with consent) will be performed. For infants who die, their placenta and internal organs will be evaluated histologically and then PCR for various organisms will be performed. Cord blood samples will undergo PCR testing for various organisms. Because of cost, these tests will only be performed in cases of neonatal death.

### Infectious causes of stillbirth and neonatal death among preterm infants

Infections are potentially a major and largely under-reported contribution to stillbirth and neonatal deaths among preterm births [14, 15]. Since the identification of pathogens responsible for fetal or neonatal death may not be possible from fetal blood, it is important to assess tissues such as the placenta, and fetal or neonatal organs.

Routine tests (related to infection) for the study will include the following for all preterm infants as clinically indicated at each of the study hospitals:

- Complete blood count (CBC)
- C-reactive protein (CRP)
- Placental histology (collected at delivery but analyzed for deaths only)

Clinical investigations to be obtained by the clinical staff when an infection is suspected include:

- Blood cultures for suspected sepsis
- CSF analysis and culture for suspected meningitis
- Chest x-ray for suspected pneumonia
- Urine culture for suspected urinary tract infection

### General approach for infections

In addition to the routine tests for deceased infants, MITS will be performed for most cases. Through MITS methodology we will obtain tissues from the, lung, liver, brain and potentially other organs. In addition, we will collect placental samples and fetal or neonatal and maternal blood for research investigation of infection.

We will test all available samples from all stillbirth and neonatal death cases using a real-time PCR format for selected organisms. The final selection of pathogens and methodology will be reviewed with other cause of death studies to help ensure that the results can be interpreted across currently funded initiatives to determine cause of death in LMIC [16]; however, organisms of special interest are outlined below.

#### Syphilis

Syphilis, an important cause of both stillbirth and neonatal death in some regions, will be documented for this study*.* Since syphilis is virtually always transmitted transplacentally from the mother to the fetus during pregnancy and is associated with a maternal antibody response [16, 17], all cases of syphilis should be identifiable by antibodies in maternal blood or alternately, cord or neonatal blood. For all stillbirths and neonatal deaths, we will aim to test maternal serum samples for syphilis.

#### GBS

There are two objectives for GBS in this study. The first is to determine the prevalence of GBS carriage in the mothers that will be determined through culture evaluation of a rectovaginal swab collected upon hospital admission for women admitted with a stillbirth or in preterm labor, with preterm rupture of membranes or with a medical condition such as preeclampsia requiring preterm delivery. Our second related objective is to assess GBS as a cause of stillbirth and neonatal death, assessed through evaluation of tissue samples collected at autopsy or by MITS and assessed by PCR.

#### Acinetobacter

*Acinetobacter* infection has been described as a cause of perinatal death, especially in very preterm babies and in chorioamnionitis associated with preterm birth. *Acinetobacter* has been described as an important contributor to neonatal infection in several LMIC reports [18–20]. While we suspect that the origin of many fetal or neonatal infections is often an intrauterine infection which led to the preterm birth, *Acinetobacter* also may be acquired by the infant in the NICU due to contaminated equipment [18]. The organism will be identified with the PCR tests. As with GBS, we will evaluate all available fetal and neonatal tissues and the placentas for *Acinetobacter* in neonatal deaths and stillbirths.

#### Respiratory syncytial virus (RSV) and other respiratory viruses

RSV, a known viral pathogen in the neonatal period, and other respiratory viruses also contribute to neonatal deaths [21] and potentially stillbirths. To evaluate the association of RSV and other respiratory viruses as a cause for both stillbirth and neonatal death, we will analyze available samples by PCR for RSV to determine its contribution to mortality.

#### *Cytomegalovirus* (CMV)

CMV, a major infectious cause of congenital abnormalities in the fetus and newborn, also causes stillbirths and neonatal deaths [22]. Most primary maternal CMV infections are clinically silent and undetected. Therefore, the placenta, blood and fetal/infant tissues will be evaluated for CMV as a cause of death. As with the other infections, using PCR, we will analyze all available samples for CMV to determine its contribution to mortality.

#### Ureaplasma and mycoplasma

*Ureaplasma* and *Mycoplasma* spp., common commensals found in the vagina, have been associated with preterm birth, neonatal infection and stillbirth [22]. These organisms can cross the amnion and chorion into amniotic fluid even with intact membranes. In a study performed by Goldenberg et al. in Alabama, *Ureaplasma* and *Mycoplasma* were frequently cultured from cord blood of preterm infants displaying a correlation between gestational age and culture positivity [23]. Both organisms can be detected by PCR. We will conduct PCR evaluations on the tissues of all stillbirths and neonatal deaths to assess the role of *Ureaplasma* and *Mycoplasma* in preterm mortality and stillbirth.

### Maternal colonization and neonatal/fetal infection

In addition to the select pathogens noted above, we will also test for other organisms known or suspected to cause stillbirth or neonatal death. Maternal colonization with pathogens including *E. coli, Pseudomonas, Staphylococcus aureus*, and *Klebsiella* have been associated with early-onset sepsis in preterm infants and cause of death in stillbirths. These organisms will be identified in the cord blood, placenta, and tissues taken at the time of MITS/autopsy in neonatal deaths and stillbirths by PCR. Other organisms such as *Fusiforme nucleatum*, an oral commensal, and other oral bacteria, such as *Campylobacter* spp. are associated with perinatal death, but have not been routinely investigated as causes of perinatal death in LMIC.

Finally, pathogens such as Lyme disease or other pathogens are known causes of stillbirth and their importance as a cause of death in South Asia is not clear [9]. Because we will have serum and tissue specimens for serology and PCR, we will explore whether Lyme disease and other pathogens are an important cause of stillbirth and neonatal death in LMIC.

### Determining infection as a cause of death

We recognize that presence of an organism by itself does not indicate that the organism was causal for the stillbirth or neonatal death. To make a final determination of infection as cause of death, we will consider the PCR results together with other clinical signs of infection. Histopathology results in the placenta and fetus or neonate, white blood cell counts, and CRP results as well as the presence of organisms will allow us to designate infection as a cause of death. The PCR results will help elucidate relatively how much pathogen DNA was present.

### Congenital anomalies

Structural anomalies will be identified through several methods: ultrasound prior to delivery, visual inspection at birth, during neonatal care, or at autopsy, and potentially by x-ray evaluation. Photographs of infants and stillbirths who appear to have a genetic syndrome will be evaluated by a dysmorphologist. For those with a suspected congenital anomaly disorder, karyotyping for these deaths will be performed.

### Hemoglobinopathies

Routine screening for important hemoglobinopathies including sickle cell anemia, other inherited abnormal hemoglobin such as hemoglobin C and E, and various forms of decreased hemoglobin production such as the thalassemia is generally not carried out in the study hospitals.

We will collect a dried blood spot (DBS) card that will be available for testing of hemoglobinopathies, as needed. During the study, we will evaluate each infant who dies with a very low hemoglobin or hematocrit level or with hydrops for a hemoglobinopathy using hemoglobin electrophoresis.

Stillbirths will also have a hemoglobin or hematocrit level performed on cord or heart blood, when feasible. Those with a low hemoglobin or hematocrit level or evidence of extramedullary hematopoiesis or hydrops will have a hemoglobin electrophoresis performed.

### Metabolic disorders

Virtually all infants born in the US are screened for a panel of metabolic disorders including hypothyroidism. These analyses are performed on DBSs collected at birth. While these conditions are more often related to neurodevelopmental conditions as the infant matures, stillbirth and neonatal death associated with metabolic disorders have been described. To evaluate these conditions, we will have the DBSs available for analysis for all liveborn, preterm infants and we will test any infant who appears likely to have a metabolic disorder.

### Specimen collection and storage

Procedures performed to obtain tissue (e.g., liver, lung, brain) and non-tissue samples (e.g., blood, CSF, and vaginal swabs) from the fetus or neonate, and the mother will be executed using stringent techniques to minimize contamination. Storage of all specimens at − 80 °C will maintain long-term integrity of both microbes and molecular components (e.g., DNA, RNA, and proteins). Samples will be kept at study sites.

For tissues acquired through MITS or autopsy following stillbirth or neonatal death, multiple specimens from the same organ will be collected and divided for two different storage conditions: flash-frozen for bacterial growth and/or molecular testing (i.e. PCR), and formalin-fixed for histological analysis.

A hierarchy for downstream processing of fetal or neonatal blood will be defined. Since blood volumes will be limited, we will prioritize the allocation of blood for culture and molecular testing, since the former requires an adequate volume for reliable results. Additionally, we will use DBS cards for fetal or neonatal blood collection.

### Cause of death determination

The classification systems will use data collected prospectively among women who experience a stillbirth or deliver a preterm neonatal death. Data will be collected in a uniform manner across the study sites.

We will define criteria for designating a condition as a cause of preterm neonatal death or stillbirth (i.e., the criteria to classify a neonatal death as caused by infection, asphyxia, IVH, NEC, or RDS, or a stillbirth by conditions such asphyxia with the obstetric or fetal antecedents noted, infection, congenital anomaly). The cause of death assignments will be consistent with the World Health Organization (WHO) Internal Classification of Death (ICD-10) [24]. A panel of experts representing pediatrics and obstetrics as well as other disciplines such as pathology, microbiology and radiology, working within the framework described above, will determine the cause of death in each of the sites.

Two reviewers will be assigned to review data from each death and determine the primary and contributory causes. If the panel members disagree, a third member will review the case. Reviewers will then discuss any discrepancies in the classification of primary and contributing causes of death. The primary cause of death is defined, in accordance with the ICD, as the underlying cause of death or the disease or injury which initiated the train of morbid events leading directly to death, or the circumstances of the accident or violence which produced the fatal injury [24]. Contributing causes of death will be conditions which adversely affected the development of the condition leading to the death or stillbirth. Contributing causes are an important consideration as they may be modifiable factors, amenable to interventions.

### Statistical analysis and sample size

We estimate that evaluating 350 preterm neonatal deaths in each site for a total of 700 preterm neonatal deaths will provide informative data about preterm neonatal deaths in each site and be representative of preterm mortality in South Asia. Assuming a mortality rate of 15% among preterm infants, to evaluate 700 deaths, each site will need to recruit approximately 2500 preterm live born infants. In addition, we will enroll the 700 women with stillbirths over the same period.

**Fig. 1:**
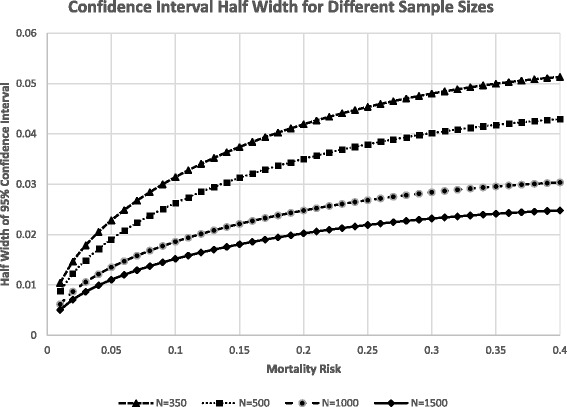
Confidence Interval (CI) to estimate cause of death given proportion of deaths

For the sample size, our main objective is to estimate the proportion of deaths by cause with a reasonably sized confidence interval around that proportion. Fig. 1 illustrates the proportion of deaths attributed to any given cause (i.e., RDS). The vertical axis shows the confidence interval, and the line shows the confluence of the proportion of deaths and confidence interval (CI) half width for a given proportion of deaths. For example, if RDS caused 20% of neonatal deaths and there are 500 neonatal deaths, the Cl width would be about 0.075. If 40% of stillbirths were the result of asphyxia and there were 500 stillbirths, the width of the CIs would be about 0.09. We chose 350 evaluable neonatal deaths and 350 stillbirths for each site as a reasonable compromise between higher CIs and study practicality including resources, and timeframe to complete study recruitment.

## Discussion

This prospective study on cause of death among stillbirths and preterm neonatal deaths will be among the first to complete in-depth evaluation of deaths in South Asia, a region which contributes the highest number of deaths globally. One of the study’s limitations is that to collect complete data, we are only able to include births that occur in the study hospitals. However, with the increasing rates of hospital deliveries in Asia, we think the impact on the study’s generalizability will be minimal. We will also have less information for infant deaths that occur during the neonatal period but outside of a study facility, which will make the performance of an autopsy and MITS difficult.

However, even with these potential limitations, this study will more clearly define the role of infectious pathogens will be more clearly defined for these settings, in which most deliveries are now occurring in hospitals. This study will augment other efforts to define the contribution of preterm birth and stillbirth to perinatal mortality. Determining the main risk factors and causes for perinatal mortality will ultimately inform potential strategies to reduce the high neonatal mortality and stillbirth rates currently seen in South Asia.
